# Effect of *Azadirachta indica* and *Senna siamea* Decoction on CD4+ and CD8+ Level, Toxicological, and Antioxidant Profile in HIV/AIDS Positive Persons

**DOI:** 10.1155/2021/5594505

**Published:** 2021-06-23

**Authors:** Oumarou Goni Hamadama, Mbah Ntepe Leonel Javeres, Nyunaï Nyemb, Medou Mba Fabrice, Pettang Tomen Manuela Elsa

**Affiliations:** ^1^Institute of Medical Research and Medical Plants Studies, Yaounde, Cameroon; ^2^Falcuty of Health Sciences, University of Yaounde 1, Yaounde, Cameroon

## Abstract

Acquired immune deficiency syndrome (AIDS) is a major public health problem affecting several countries with predominance in black Africa. Faced with therapeutic failure caused by resistance and supply disruptions, searching for other antiretroviral agents, in particular from natural sources, becomes necessary. Given popular consumption of *Azadirachta indica* and *Senna siamea* decoction in the Northern Cameroon region and the traditionally attributed antiretroviral value, information on its efficacy and safety consumption is relevant to confirm its use. A total of 297 participants aged 18–52 and HIV-positive were recruited and divided into 3 groups: one taking only the decoction (group 1), another taking only antiretroviral therapy (ARTs) (group 2), and finally, one taking the decoction and antiretroviral (group 3). During 6 months, all the participants of the concerned groups consumed daily (morning and evening) 250 mL of *Azadirachta indica* and *Senna siamea* decoction. CD4+ and CD8+ levels were measured by flow cytometry. Hepatic and renal toxicity and oxidative stress were evaluated spectrophotometrically by measuring ALT, AST, ALP, BUN, CREAT, SOD, CAT, and GSH parameters. We note an increase in the CD4+ level of the three groups with values much more pronounced in the group treated by ARTs + decoction, from 328 ± 106 to 752 ± 140. Group 2 presented not only biological signs of hepatic and renal toxicity but also significant oxidative stress. No signs of toxicity were detected in the other groups. The study concludes that a decoction of *Azadirachta indica* and *Senna siamea* stimulates the production of CD4+ and is not toxic. On the contrary, it would reduce the toxicity caused by ARTs intake.

## 1. Introduction

Acquired immune deficiency syndrome (AIDS), caused by the human immunodeficiency virus (HIV), has been a major public health problem of global concern for several years. AIDS since its onset has already caused more than 32 million deaths worldwide. According to the World Health Organization, the total number of people living with HIV/AIDS in 2019 was approximately 38.0 million (31.6 million–44.5 million) worldwide. Africa is particularly affected by this pandemic with more than two-thirds of the number of cases or 25.7 million of these people. In Cameroon, HIV prevalence among adults was 3.6% in 2019: 5.0% among women and 2.3% among men. This corresponds to approximately 540,000 adults living with HIV in Cameroon [1].

HIV/AIDS is managed by taking antiretroviral therapies (ARTs), which are used in various combinations commonly known as highly active retroviral therapy (HAART). Treatment with HAART promotes a sustained decrease in viral load and restoration of the immune response, as well as a reduction in morbidity and mortality from HIV infection [2]. However, its long-term use is limited by metabolic disturbances, toxicities, and the emergence of drug-resistant viruses [3, 4].

Since 2019, taking of ARTs is systematic in HIV/AIDS patients regardless of the clinical status and CD4+ level. Expansion of treatment access has led to several problems such as the unavailability of antiretrovirals, difficulties in laboratory monitoring, and an increased rate of treatment failure [5]. A study carried out in Cameroon in positive children showed that treatment failures and transition to the second line depended among other things on noncompliance with treatment and immunological status [6, 7].

Faced with the therapeutic failure caused by resistance and the break in taming of ARTs, the search for new antiretroviral agents more effective and with low toxicity, in particular from natural sources, becomes urgent.

Natural resources such as medicinal plants continue to be a common alternative for treating a variety of infectious and noninfectious diseases. It has been widely documented that medicinal plants are being used to treat HIV/AIDS with limited or no side effects [8]. Due to the potential source of antioxidants and nutraceutical compounds, medicinal herbs not only affect viral particle replication but also function as immunomodulators and immune stimulants. There have been several reports of herbs with anti-HIV activity in literature [9].

An ethnobotanical study conducted in sub-Saharan Africa indicated that traditional healers and local communities widely use medicinal plants to manage the effect of HIV/AIDS in Cameroon [10].

Thus, there is a need to discover new anti-HIV agents to supplement our current arsenal of anti-HIV drugs and to provide therapeutic options for populations with limited resources or access to currently efficacious chemotherapies. Plant-derived natural products continue to serve as a reservoir for the discovery of new medicines, including anti-HIV agents.

In the North Cameroon region, two medicinal plants, *Azadirachta indica* (neem), and *Senna siamea*, are widely used for HIV/AIDS treatment in the community. The therapeutic virtues of *Azadirachta indica* and *Senna siamea* are strongly referenced with several activities and effects attributed to them.

Alzohairy reports several pharmacological properties of neem which have already been demonstrated, amongst which are anticancer, immunomodulatory, antidiabetic, neuroprotective, anti-inflammatory, antiviral, antibacterial, antifungal, antioxidant, antimalarial, hepatoprotective, antinephrotoxic, and wound healing activities. However, the various studies evaluating the toxicity of neem remain controversial because some attribute toxicity to neem while others have shown its harmlessness [11].

Clinical trials have also shown that the extract of *Azadirachta indica* leaves inhibits the invasion of human lymphocytes by HIV-I and causes a significant improvement in CD4+ cell number in a small number of patients with HIV/AIDS [12].

A double-blind clinical drug trial revealed that patients taking the drug made up of aqueous extract of neem leaves in addition to coal tar had shown a quicker and better response in comparison to the placebo group [11]. Another clinical study of six weeks showed that the dental gel containing neem extract significantly reduced the plaque index and bacterial count compared to that of the control group [11].

On the other hand, Kamagaté et al. report in a review that *C. siamea* has antimicrobial, antimalarial, antidiabetic, anticancer, hypotensive, diuretic, antioxidant, laxative, anti-inflammatory, analgesic, antipyretic, anxiolytic, antidepressant, and sedative activities [13]. Although many studies have already reported the antiretroviral activity of *A. indica*, no studies have corroborated this activity for *Senna siamea.* Likewise, and to the best of our knowledge, the antiretroviral effect of the combined intake of these two plants is not known. Even more, the risks of drug interactions with conventional treatments, especially with ARTs, have never been evaluated.

Considering the popular consumption of the decoction of these two plants in the North Cameroon region and the traditional medicinal value credited, their real properties must be evaluated to confirm their use. Our study aims to not only assess the antiretroviral potential of the decoction of the 2 plants but also to assess this antiretroviral potential when the decoction is coupled with HAART.

## 2. Methodology

### 2.1. Study Population, Decoction Preparation, and Blood Collection

Participants were recruited at the approved HIV/AIDS Treatment Center (CTA) of the Garoua-Nord-Cameroon Regional Hospital and from phytotherapists residing in the city of Garoua and using the decoction of *Azadirachta indica* and *Senna siamea* for HIV/AIDS treatment. Ethics approval was obtained from the Ethical Review Board of the University of Yaoundé I, Cameroon (UY1/COET/EM/0190/19). The study confirmed to tenets of the Helsinki Declaration for human subjects in experiments. All the study participants were informed about the purpose of the study, and written consent was taken before sample collection.

The participants were in the age group of 18–52 years, HIV-positive, and living in the collection areas. Subjects were excluded if they disclosed having other diseases such as diabetes, neurological disorders, liver dysfunction, renal dysfunction, cancer, or any other chronic condition. Participants were then classified into three groups. The first group consisted of HIV patients taking only the decoction as treatment. The second group consisted of HIV patients taking only antiretroviral therapy. The third group consisted of HIV patients taking the decoction plus antiretroviral therapy as treatment. The patients on ART were all on the first therapeutic line which constituted of ARTs tenofovir (TDF), lamivudine (3TC), efavirenz (EFV), or nelfinavir (NFV). Questionnaires were provided to collect demographic characteristics (age, sex, height, weight, profession, and marital status).

The sample size was calculated using the Lorentz formula, based on the prevalence of HIV in Cameroon (3.8%, CAMPHIA [14]). The samples were increased to 10% (to keep statistical power in case of selection bias). After screening and exclusion, a total of 297 participants were selected to participate in the study, i.e., 97 people taking only the decoction (group 1), 100 people taking only the ARTs (group 2), and 100 taking the decoction plus ARTs (group 3). During 6 months, all the participants of the concerned groups consumed daily (morning and evening) 250 mL of *Azadirachta indica* and *Senna siamea* decoction, ART, or a combination of ART + decoction.

Although the decoction is widely used in the community, for this study, it was directly prepared by the herbalist by boiling in 5 L of water for 15 minutes approximately 0.5 kg of *Azadirachta indica* leaves and 0.5 kg of *Senna siamea* leaves.

### 2.2. Phytochemical Screening

The phytochemical analysis was carried out as described previously by Harborne [15]. Based on the color intensity of the solutions thresholds: absence (−), trace (+/−), weak (+), abundant (++), and very abundant (+++) were used to qualitatively define and quantitatively estimate the intensity of each metabolite in plants.

### 2.3. Blood Collection

Blood samples were taken from all participants using aseptic venipuncture and transported to the laboratory in ice bags. Four series of samples were taken. For toxicological analyses, samples were collected on day 0, after 1 month, and after 3 months. 6 months after, samples were collected for immunological analyses. The collected blood was divided into two tubes for further analysis of plasma and serum fractions, using EDTA-coated glass and plain glass tubes, respectively. The blood was centrifuged at 3500 rpm for 10 minutes at 25°C. The plasma and serum thus obtained were then stored at −80°C for subsequent analysis. The samples were analyzed at the IMPM Laboratory, Yaoundé, Cameroon, and the Center Pasteur Annex in Garoua, Cameroon.

### 2.4. Biological Analysis

#### 2.4.1. CD4+ and CD8+ Cell Count

On day zero and after six months, 3 mL of blood taken from an EDTA tube was introduced into an automated device (BD FACSCount ™ system) to measure CD4+ and CD8+ in the absolute value. The cell counting method used by our automaton was based on flow cytometry as previously described [16].

### 2.5. Determination of Biochemical Parameters

Creatinine (CREAT), blood urea nitrogen (BUN), aspartate transaminase (AST), alanine transaminase (ALT), and alkaline phosphatase (ALP) were determined with commercial kits, by enzymatic tests according to the manufacturers' protocols supplied by the kits from BIOLABO SA, Les Hautes Rives, 02160, Maizy, France. Optical densities were determined using the Kenza Automatic Biochemistry Analyzer, 450TX.

### 2.6. Measurement of Antioxidant Parameters

Glutathione reductase (GSH) was determined by the method using Ellman reagent [17]. Measurement of superoxide dismutase (SOD) was carried out by spectrophotometry according to the methods of Misra and Fridovich [18], and the measurement of catalase was carried out following the method for evaluating the transformation of dichromate-acetate described by [19].

### 2.7. Statistical Analyses

Stata 15 and R 3.2.0 softwares were used for the statistical analysis of data. The normality of the distribution of variables was assessed using the Shapiro–Wilk test. One-way ANOVA with posthoc Tukey was carried out for comparison of hepatic, oxidative stress, and immunological parameters of different treated groups. A logistic regression model was used to establish a relationship between the different treated groups and explanatory variables (biochemical and immunologic parameters). Odds ratios (OR) with 95% confidence intervals (95% CI) were calculated. The differences were considered statistically significant at *p* < 0.05.

## 3. Results

### 3.1. Sociodemographic Characteristics

Population distribution according to the sociodemographic characteristics shows that the majority of participants (60%) were between 30 and 45 years of age, average age was 35.64 years. The sex ratio (male/female) was 2.25 (Table 1).

### 3.2. Phytochemicals Screening

Phytochemical analysis reveals the abundant presence of phenolic compounds in our two extracts. It also noted the presence of a high quantity of flavonoids in *Azadirachta indica* extract and coumarins in *Senna siamea* extract (Table 2).

### 3.3. Immunologic Parameters

After 6 months of treatment, we note an increase in CD4+ count in all three groups. This increase was much more pronounced in the group treated with ART + decoction, from 328 ± 106 to 752 ± 140 against 357 ± 122 to 549 ± 217 in the group treated only with ART. CD4+/CD8+ ratios also show a more pronounced increase in the group treated with ARTs +0.73 ± 0.33 decoction (Table 3).

### 3.4. Toxicological Parameters

After 30 days of treatment, evaluation of hepatic and renal function as well as oxidative stress shows that group 2 participants (ART only) had much higher activity levels of AST and ALP than group 1 participants (decoction only) and group 3 participants (combination ART + decoction). We also note a significant decrease in CAT and GSH in the ART group compared to the other groups. After 3 months of treatment, only the creatinine remained the same. The rest of the parameters became even higher in group 2 (ART only group). There was also a significant decrease in SOD in the same group (Table 4).

**Figure 1:**
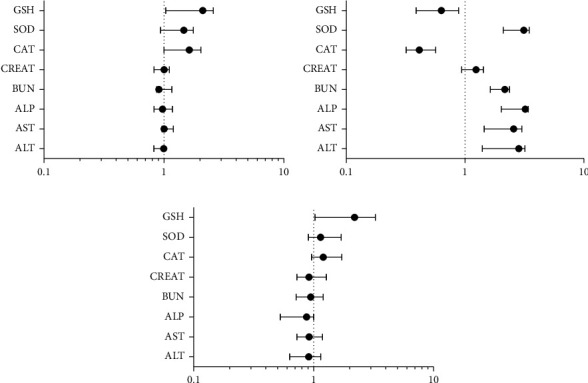
Binomial logistic regression of biochemical parameters as a time function. The model was adjusted for each graph (0, day zero of treatment; 1, after 3 months of treatment). The results are significant if *p* < 0.05. (a) Odds ratio of the decoction group. (b) Odds ratio of the ARV group. (c) Odds ratio of the ART + decoction group.

In group 1 (decoction), only a few parameters undergo a significant increase over time (from day 0 to the end of the 3rd month). These increases include, among other parameters, catalase (OR: 1.627; CI: 1.002–2.034), SOD (OR: 1.467; CI: 0.936–1.764), and GSH (OR: 2.109; CI: 1.034–2.589) (Figure 1(a)). In group 2 (ART), there was a significant increase in all hepatic and renal parameters. A significant decrease in CAT and GSH was also recorded (Figure 1(b)). In group 3 (ART + decoction), only GSH (OD: 2.197; CI: 1.027–3.274) undergoes an increase. The rest of the parameters remained unchanged (Figure 1).

## 4. Discussion

Until now, HIV/AIDS has represented a real challenge for sub-Saharan African countries such as Cameroon. In these countries, for economic and sociocultural reasons and sometimes because of shortages of ARTs, alternative herbal therapies are often used by HIV/AIDS patients [20]. Over the years, in the North Region of Cameroon, the decoction of *Azadirachta indica* and *Senna siamea* has been widely consumed in the community, and many attributes its retroviral potential.

The clinical and biological manifestations observed during HIV infection are due to the massive destruction of CD4+ lymphocytes. Several mechanisms are implemented to lead to the depletion of this cell population. These include the cytopathogenic effect of viruses, cytotoxic immune responses, apoptosis, and also loss of regenerative capacity of these cells by the lymphoid system [21].

During this study, we determined the plasma concentrations of CD4+ and CD8+ lymphocytes and the CD4+/CD8+ ratio of included subjects to assess their immune capacity. Our results reveal a significant increase in the level of CD4+ in all the groups of participants after 6 months of treatment. This increase is much more significant in the group treated with ART + decoction (group 3) in comparison with the other groups (group 2, ART only and group 1, decoction only). CD4+/CD8+ ratios also show an increase in all groups but remain below 1. This increase follows the same pattern as the rate of CD4+, higher in the group treated with ART + decoction. Although this decoction is highly consumed in North Cameroon, no previous scientific report has studied the effect of the consumption of this decoction on health in general and in HIV-positive patients in particular. The literature, however, is full of previous observations on *Azadirachta indica* and its antiretroviral properties. Mbah et al. (2007) in a 12-week study showed that extracts of *Azadirachta indica* leaves administered (1 g per day) to 60 infected patients could increase the average number of CD4+ by 266 cells/*μ*l over the study period [22]. In 2009, Anyaehie compared the antiretroviral properties of *Azadirachta indica* leaf extracts with those of highly active antiretrovirals (HAART). These results show a greater increase in CD4+ cells in patients on HAART compared to patients on *Azadirachta indica* [23]. Mbah et al. and Anyaehie therefore agree, as is also the case in our study, that there was a significant improvement in the immune system, after consumption of *Azadirachta indica* extract. When compared with previous studies, the immunostimulatory effect of the decoction of *Azadirachta indica* and *Senna siamea* is much greater. This difference could be explained by the fact that this decoction is a mixture of two plants. One with an antiretroviral-HIV activity already proven (*Azadirachta indica*) and the other (*Senna siamea)* having a good antioxidant activity, and probably, also an antiretroviral-HIV activity which, however, has never been evaluated before. The antiretroviral mechanisms of this extract remain very controversial, however. One of the most likely mechanisms is that active ingredients in plants could inhibit syncytia formation by binding to viral gp120 protein, blocking gp120-CD4+ interaction, thereby not only inhibiting HIV binding to CD4+ cells but also preventing syncytium formation (giant cell) induced by HIV [12, 23].

In HIV/AIDS infection context, studies have shown that lymphocytes die of apoptosis and that all subpopulations are affected: not only CD4+ but also CD8+, monocytes, and B cells. It has also been shown that taking ARTs can be toxic to the liver and kidneys and increased oxidative stress in HIV + patients [24, 25]. Its oxidants accelerate the degradation of the immune system, including a decrease in lymphocytes, and may even help stimulate viral replication. This phenomenon is amplified by the deficit in antioxidants. Indeed, Tat protein has the ability to reduce the expression of superoxide dismutase and glutathione peroxidase genes, which are key enzymes in an antioxidant response [22, 26–28].

In this study, from the first month of treatment and until the 3^rd^ month, group 2 (ART only) presented not only biological signs of hepatic and renal toxicity but also significant oxidative stress. On the other hand, no sign of toxicity was detected in group 1 (decoction only) and group 3 (ART + decoction). These results lead to the belief that consuming the extract is not only safe over 3 months (group 1 results) but also that the decoction could have a beneficial effect by detoxifying ARTs activity (comparing results of groups 2 and 3). This property of the decoction could be due to the antioxidant effect of different plants.

Transaminase activity is high in the group of patients treated only with ARTs, while its activity level remains the same for groups 1 and 3, both after 30 days and after 3 months. This same profile is also observed with ALP. Elevation of transaminases indicates impaired liver function or cytolysis [29, 30]. Elevations of liver enzymes often reflect damage to the liver or biliary obstruction. Alanine aminotransferase (ALT), aspartate aminotransferase (AST), alkaline phosphatase, and bilirubin are biochemical markers of liver injury. Hepatic enzyme elevations are normal in HIV patients, especially those on antiretroviral therapy (ART). Despite these statistics, HIV-positive patients can have many risk factors for biochemical disorders, although the exact cause is rarely determined [31]. The majority of hepatotoxicity studies have shown that ART causes hepatic damage, but only in the presence of other factors such as HCV [32] and even tuberculosis [33]. A rise in ALP may also indicate biliary obstruction.

As a result of our findings, the groups treated with ART alone had lower CAT and GSH levels than the other groups. This deterioration could lead to oxidative stress, resulting in damage to cells and membranes in the renal tissue. The reversed levels after treatment with an extract or its coadministration with ART, on the other hand, suggest an antioxidative therapeutic effect against ART-induced oxidative stress through substantial enhancement of enzymatic antioxidant activities. These results are similar to those previously obtained by Zhang et al. [34].

After 3 months of treatment, the urea level in group 2 of people treated only with ARTs is high. BUN and serum CREAT are commonly used as markers for normal biological, pathologic, or pharmacologic responses to clinical practice as markers of renal function. In ART-treated patients, we discovered substantially elevated BUN levels which can be an indicator of progressive substantial impairment in renal function. These increases in BUN concentration could have resulted from significant leakage caused by glomerular hypercellularity and tubular degradation. Treatment with the decoction significantly reduced kidney injury markers, such as BUN, preventing the development of nephrotoxicity. The attenuation of the increase in BUN in group 3 supports the activity of the biologically active components of the plant.

Indeed, among the many properties of our plants, *Azadirachta indica* and *Senna siamea* also have a very good antioxidant power, which would allow them to reduce the damage induced by oxidative stress [26, 28, 30]. Xiang et al. demonstrated that *Azadirachta indica* significantly increased the levels of CAT and GSH [22]. Likewise, Olutayo et al. suggested that *Azadirachta indica* considerably attenuates oxidative stress and inflammation [35]. Gupta et al. showed that *Azadirachta indica* significantly increased the levels of CAT and SOD and decreased the levels of GSH [30]. Barnaby et al. in a study on the antioxidant power of several plants demonstrated that *Senna siamea* had a very high antioxidant power [28]. This antioxidant power could be related to the presence of secondary metabolic agents such as flavonoids and other phenolic compounds, such as gallic acid known for their antifree radical and/or antioxidant properties [30, 36, 37].

## 5. Conclusion

A decoction of *Azadirachta indica* and *Senna siamea* boosts the immune system of HIV-positive patients, by stimulating the production of CD4+ cells. Daily consumption of the decoction over 3 months is not toxic; on the contrary, it would reduce the liver and kidney toxicity caused by taking antiretroviral therapy (ARTs), by increasing the production of antioxidant enzymes, such as SOD and catalase.

### 5.1. Limitations and Perspectives

There are certain limitations to the study that should be mentioned here. For example, it would be essential that further studies be devoted to the antiretroviral activity of *Senna siamea* only which until now has never been realized. Similarly, it is necessary to fractionate these plants to identify the metabolites responsible for these effects. Late chronic toxicity studies are also required to rule out the possibility of any delayed toxic effects.

## Figures and Tables

**Table 1:** Characteristics of the study population.

Characteristics	Total population (*N* = 297)
Age	
Age (years) mean ± SEM	35.64.22 ± 10.12
18–29	25 (75)
30–45	60 (179)
+45	15 (43)

Gender % (*N*)	
Male	69.02 (205)
Female	30.98 (92)
Sex ratio (male/female)	2.25

*N* = total number of samples.

**Table 2:** Phytochemical screening of each extract of the decoction.

Chemical groups	Plants extracted (leaves)	
	*Azadirachta indica*	*Senna siamea*
Phenolic compounds	+++	+++
Flavonoids	+++	+
Glucosides	−	+
Steroids	+	−
Triterpenes	++	+
Saponosides	−	+
Coumarins	−	+++
Tannins	+	−
Catechin	+	+
Alkaloids	+/−	+/−

−, absence; +/−, traces; +, low presence; ++, abundant; +++, very abundant.

**Table 3:** Variation of LT CD4+ and LT CD8+ in different groups.

Period	Parameters	Group 1 *N* = 97	Group 2 *N* = 100	Group 3 *N* = 100	*P* value	ODD
Day 0	CD4+	381 ± 12	357 ± 13	328 ± 11	0.4	0.973 (0.742–1.104)
	CD8+	1382 ± 49	1279 ± 56	1373 ± 42	0.1	0.871 (0.714–1.001)
	CD4+/CD8+	0.27 ± 0.03	0.28 ± 0.02	0.24 ± 0.02	0.1	1.028 (0.804–1.111)

After 6 months	CD4+	501 ± 24^a^	549 ± 22^a^	752 ± 14^b^	0.01	1.634 (0.931–2.042)
	CD8+	1407 ± 47	1007 ± 56	1026 ± 41	0.1	1.004 (0.847–1.135)
	CD4+/CD8+	0.37 ± 0.03^a^	0.54 ± 0.0.04^b^	0.73 ± 0.04^c^	0.02	1.719 (1.082–2.109)

Group 1, decoction; Group 2, ART; Group 3, ART + decoction. Results are expressed in means ± SEM; a, b, and c indicate the statistical differences of Tukey' posthoc test; statistically significant (*p* ≤ 0.05, one-way ANOVA of groups 1, 2, and 3).

**Table 4:** Variation of biochemical parameters in different groups.

Time	Parameters	Group 1 *N* = 97	Group 2 *N* = 100	Group 3 *N* = 100	*P* value
Day 0	ALT (UI/L)	37 ± 9	35 ± 12	40 ± 8	0.123
	AST (UI/L)	34 ± 8	39 ± 9	36 ± 7	0.280
	ALP (UI/L)	94 ± 14	102 ± 17	89 ± 12	0.178
	BUN (mmol/L)	10 ± 3	10 ± 4	9 ± 3	0.523
	Creatinine (*μ*mol/L)	77 ± 12	74 ± 10	80 ± 17	0.347
	CAT (U/mg protein)	0.407 ± 0.103	0.367 ± 0.120	0.480 ± 0.103	0.157
	SOD (U/mg protein)	10.14 ± 3.20	11.96 ± 2.36	10.58 ± 3.34	0.309
	GSH (*μ*mol/mL)	16.55 ± 5.13	16.57 ± 5.39	18.82 ± 5.74	0.291

After 30 days	ALT (UI/L)	30 ± 10	55 ± 16	46 ± 9	0.214
	AST (UI/L)	29 ± 5^a^	54 ± 14^b^	34 ± 6^a^	0.04
	ALP (UI/L)	100 ± 27^a^	230 ± 71^b^	94 ± 13^a^	0.01
	BUN (mmol/L)	10 ± 2	16 ± 5	10 ± 4	0.127
	Creatinine (*μ*mol/L)	82 ± 19	110 ± 28	71 ± 16	0.08
	CAT (U/mg protein)	0.615 ± 0.106^c^	0.247 ± 0.113^a^	0.510 ± 0.124^b^	0.01
	SOD (U/mg protein)	11.10 ± 3.07	20.96 ± 7.15	16.34 ± 4.07	0.06
	GSH (*μ*mol/mL)	36.75 ± 6.24^b^	10.57 ± 3.14^a^	24.80 ± 5.40^b^	0.03

After 3 months	ALT (UI/L)	33 ± 6^a^	91 ± 23^b^	42 ± 8^a^	0.03
	AST (UI/L)	36 ± 10^a^	84 ± 27^b^	36 ± 4^a^	0.01
	ALP (UI/L)	108 ± 35^a^	327 ± 101^b^	100 ± 16^a^	0.01
	BUN (mmol/L)	8 ± 3^a^	27 ± 9^b^	10 ± 4^a^	0.04
	Creatinine (*μ*mol/L)	79 ± 17	114 ± 41	76 ± 14	0.07
	CAT (U/mg protein)	0.679 ± 0.121^b^	0.252 ± 0.109^a^	0.564 ± 0.119^b^	0.01
	SOD (U/mg protein)	15.50 ± 3.43^a^	38.76 ± 13.23^b^	17.86 ± 4.12^a^	0.03
	GSH (*μ*mol/mL)	34.39 ± 8.40^b^	12.80 ± 4.12^a^	47.63 ± 10.02^b^	0.01

Group 1, decoction; Group 2, ART; Group 3, ART + decoction. AST, aspartate transaminase; ALT, alanine transaminase; ALP, alkaline phosphatase; BUN, blood urea nitrogen; CAT, catalase; SOD, superoxide dismutase. Results are in means ± SEM; a, b, and c indicate statistical differences of Tukey' posthoc test; statistically significant (*p* ≤ 0.05, one-way ANOVA of groups 1, 2, and 3).

## Data Availability

The data used to support the findings of this study are included within the article.
